# Structural characterization, antioxidant activity, and antiglycation activity of polysaccharides from different chrysanthemum teas

**DOI:** 10.1039/c9ra05820f

**Published:** 2019-11-01

**Authors:** Qin Yuan, Yuan Fu, Pan-Yin Xiang, Li Zhao, Sheng-Peng Wang, Qing Zhang, Yun-Tao Liu, Wen Qin, De-Qiang Li, Ding-Tao Wu

**Affiliations:** College of Food Science, Sichuan Agricultural University Ya'an 625014 Sichuan China DT_Wu@sicau.edu.cn; Department of Pharmacy, The Second Hospital of Hebei Medical University Shijiazhuang China deqli@163.com +86 835 2883219 +86 835 2883219; State Key Laboratory of Quality Research in Chinese Medicine, Institute of Chinese Medical Sciences, University of Macau Macao China

## Abstract

Polysaccharides are one of the major bioactive components in chrysanthemum teas. In order to understand well the chemical structures and bioactivities of polysaccharides from different chrysanthemum teas (JHPs) collected in China, the physicochemical characteristics, antioxidant activity, and antiglycation activity of polysaccharides extracted from different chrysanthemum teas, including *Coreopsis tinctoria*, *Chrysanthemum indicum*, *C. morifolium* ‘Huangju’, *C. morifolium* ‘Gongju’, and *C. morifolium* ‘Hangbaiju’, were investigated. The results showed that the contents of total uronic acids and total phenolics in JHPs ranged from (28.4 ± 0.3)% to (36.2 ± 0.2)%, and from 9.4 ± 0.7 to 70.2 ± 1.7 mg GAE per g, respectively. The molecular weights of fraction 1 and fraction 2 in JHPs ranged from 4.29 × 10^5^ to 5.88 × 10^5^ Da, and from 4.11 × 10^4^ to 5.24 × 10^4^ Da, respectively. The dominant constituent monosaccharides of JHPs were galacturonic acid, arabinose, and galactose. Furthermore, JHPs, especially polysaccharides extracted from *C. tinctoria*, exerted remarkable ABTS, DPPH, nitric oxide, and hydroxyl radical scavenging activities, as well as strong antiglycation activities. The results are helpful for better understanding of the chemical structures and bioactivities of JHPs, and JHPs may have good potential applications in the functional-food industry.

## Abstract 2

In this study, structural characteristics, antioxidant activity, and antiglycation activity of polysaccharides from different chrysanthemum teas were investigated and compared.

## Graphical abstract

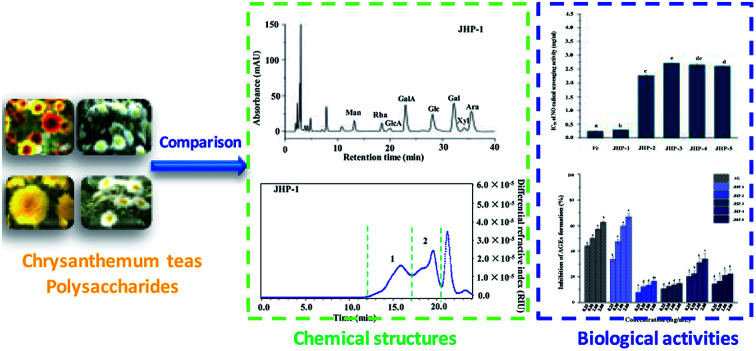

## 1. Introduction

Oxidative stress arises due to a disturbance in the balance of pro-oxidant and anti-oxidant systems in the body, which is characterized by excessive reactive oxygen species (ROS) production.^1^ Excessive accumulation of ROS results in the generation of free radicals which can cause oxidative damage of deoxyribonucleic acids (DNA), proteins, and lipids.^2^ Increasing evidence suggests that free radical induced oxidative stress plays an important role in the pathophysiology of many human diseases, such as cancer, cardiovascular disease, inflammatory diseases, and neurodegenerative disorders.^2,3^ Furthermore, glycation and oxidative stress are closely related and are often referred to as “glycoxidation” processes.^4^ Glycation is a spontaneous non-enzymatic amino–carbonyl reaction between reducing sugars and proteins followed by the formation of an early glycation product which undergoes rearrangement, dehydration and cyclization to form Schiff base and Amadori products, which lead to the formation of advanced glycation end products (AGEs).^5,6^ AGEs can result in many chronic diseases including aging, arteriosclerosis and diabetic complications.^7,8^ All glycation stages produce oxygen-free radicals.^4^ Studies have shown that the mechanism of antiglycation may be related to its antioxidant activity.^9^ Antioxidants can alleviate the oxidative stress, which is beneficial for human health. Nowadays, many synthetic antioxidants have been used in the food and medicine industry to reduce the overproduction of ROS.^10^ However, some synthetic antioxidants are restricted use due to their potential hazards to human health.^11^ Recently, polysaccharides isolated from natural resources have been noticed as novel potential antioxidants due to their low toxicity and high level of antioxidant capacities, such as free radicals scavenging and oxidative damages reducing.^12–14^ Therefore, there are increasing interests in seeking for natural polysaccharides as natural antioxidants for the prevention of oxidative damages and glycoxidation in the functional and health food industries.

The flower of chrysanthemum has been used as a popular tea material and an important traditional Chinese medicine for many years in China.^15,16^ Chrysanthemum tea is one of the most commonly daily consumed teas for Chinese consumers. Several different species and cultivars of chrysanthemum flowers, such as *Coreopsis tinctoria* (snow chrysanthemum), *Chrysanthemum indicum*, *C. morifolium* ‘Gongju’, *C. morifolium* ‘Hangbaiju’, *C. morifolium* ‘Huangju’, *C. morifolium* ‘Hangju’, *C. morifolium* ‘Huaiju’, and *C. morifolium* ‘Boju’, are consumed as the most popular tea materials in China.^15,17^ Pharmacological studies have shown that the extracts of chrysanthemum teas possess various bioactivities, such as antioxidant, anti-inflammation, antihypertensive, neuroprotective, and antidiabetic effects.^10,15,17–21^ Generally, polysaccharides, flavonoids, phenolic acids, and volatile oil are considered the main bioactive ingredients in chrysanthemum teas,^10,15,17,20,22,23^ which are responsible for their excellent antioxidant activity. Nowadays, comparison and chemical characterization of phenolic compounds (such as flavonoids and phenolic acids) in ethanol/methanol extracts from different chrysanthemum teas, such as *C. tinctoria*, *C. morifolium* ‘Gongju’, *C. morifolium* ‘Hangbaiju’, *C. morifolium* ‘Hangju’, *C. morifolium* ‘Huaiju’, and *C. morifolium* ‘Boju’, have been widely investigated.^15,20–22^ Interestingly, the chrysanthemum flower has been primarily consumed as a tea product, which is the hot water infusion of the flowers.^15^ To date, chemical structures and bioactivities of polysaccharides, which abundant exist in different chrysanthemum teas (water decoction),^10^ have seldom been compared and investigated. Therefore, investigation and comparison of physicochemical characteristics and bioactivities of chrysanthemum tea polysaccharides (JHPs) is necessary and important, which is beneficial to well understand the chemical structures and bioactivities of polysaccharides in different chrysanthemum teas, and helpful for the development of their applications in the pharmaceutical and health food industries.

In the present study, in order to well understand the chemical structures and bioactivities of polysaccharides in different chrysanthemum teas, and to further explore their applications in the pharmaceutical and health food industries, the physicochemical characteristics, antioxidant activity, and antiglycation activity of polysaccharides from different chrysanthemum teas were systematically investigated and compared.

## 2. Material and methods

### 2.1. Material and chemicals

Different chrysanthemum teas, including *C. tinctoria* (snow chrysanthemum tea), *C. indicum*, *C. morifolium* ‘Huangju’, *C. morifolium* ‘Gongju’, and *C. morifolium* ‘Hangbaiju’, were purchased from a local market in Ya'an, China. Chrysanthemum teas were dried at the temperature of 45 °C for 2 days, and then the dried samples were ground to pass through a 60 mesh sieve, and stored at −20 °C for further analysis.

Trifluoroacetic acid, monosaccharide standards (rhamnose, mannose, glucuronic acid, galacturonic acid, glucose, galactose, xylose and arabinose), 1-phenyl-3-methyl-5-pyrazolone (PMP), hydrogen peroxide (H_2_O_2_), *m*-hydroxydiphenyl, griess reagent, vitamin C, sodium nitroprusside (SNP), 2,2′-azino-bis(3-ethylbenzthiazoline-6-sulphonic acid) (ABTS), butylated hydroxytoluene (BHT), 2,2-diphenyl-1-(2,4,6-trinitrophenyl) hydrazyl (DPPH), sodium azide, bovine serum albumin (BSA), and aminoguanidine were purchased from Sigma-Aldrich (St. Louis, MO, USA). All other reagents and chemicals used were of analytical grade.

### 2.2. Preparation of polysaccharides from different chrysanthemum teas

Microwave assisted extraction (MAE) of polysaccharides from different chrysanthemum teas was preformed according to our previously optimized method.^10^ Briefly, 1.0 g of each sample was firstly refluxed twice with 10 mL of 80% (v/v) ethanol at 80 °C for 2 h to remove most of the small molecules. Then, polysaccharides from the chrysanthemum tea residue were extracted with 60.0 mL of deionized water by the microwave extraction device (MKJ-J1-3, Qingdao Makewave Microwave Applied Technology Co., Ltd., Shandong, China) at 500 W and 80 °C for 6.5 min. Furthermore, three volumes of 95% ethanol (v/v) were utilized for the precipitation of polysaccharides in the supernatant overnight at 4 °C. The precipitations were washed twice with 70% ethanol (v/v), and then were dissolved in deionized water. Then, the supernatant was dialyzed against deionized water for 48 h (dialysis membrane, molecular weight cutoff: 3.5 kDa, Solarbio, Beijing, China). Finally, the crude polysaccharides from different chrysanthemum teas (JHPs), including *C. tinctoria* (JHP-1), *C. indicum* (JHP-2), *C. morifolium* ‘Huangju’ (JHP-3), *C. morifolium* ‘Gongju’ (JHP-4), and *C. morifolium* ‘Hangbaiju’ (JHP-5), were freeze dried, and stored at −20 °C for further analysis.

### 2.3. Structural characterization of JHPs

#### 2.3.1. Chemical composition analysis

The total polysaccharides, uronic acids, and proteins contents of JHPs were determined by the phenol–sulfuric acid method using the mixture standard (40% GalA, 30% Ara, and 30% Gal) as a standard,^10^ by the *m*-hydroxydiphenyl method using GalA as a standard,^24^ and by the Bradford's method using bovine serum albumin as a standard,^25^ respectively. Furthermore, the content of total phenolics (TPC) in JHPs was determined by Folin–Ciocalteu assay using gallic acid as a reference.^26^

#### 2.3.2. Determination of weight-average molecular weights

The absolute weight-average molecular weights (*M*_w_) and polydispersities (*M*_w_/*M*_n_) of JHP-1, JHP-2, JHP-3, JHP-4, and JHP-5 were measured by high performance size exclusion chromatography coupled with multi angle laser light scattering and refractive index detector (HPSEC-MALLS-RID, Wyatt Technology Co., Santa Barbara, CA, USA) based on our previously reported method.^27,28^ The TSKgel GMPWXL (300 mm × 7.8 mm, i.d.) column was utilized for the separation of JHPs at 30 °C. The Astra software (version 7.1.3, Wyatt Technology Co., Santa Barbara, CA, USA) was utilized for data acquisition and analysis.

#### 2.3.3. Determination of constituent monosaccharides

Constituent monosaccharides of polysaccharides from different chrysanthemum teas were measured by high-performance liquid chromatography (HPLC) analysis according to our previously reported method.^10^ Briefly, each sample (4.0 mg) was hydrolyzed with 2.0 M trifluoroacetic acid at 95 °C for 10 h. Subsequently, the dried hydrolyzates were dissolved in 1 mL of water for PMP derivatization. Meanwhile, a mixed standard solution, containing Rha, Man, GlcA, GalA, Glc, Gal, Xyl, and Ara, was also derivatized. Finally, the PMP derivatives were analyzed by an Agilent 1260 series LC system (Agilent Technologies, Palo Alto, CA, USA) with a ZORBAX Eclipse XDB-C18 column (4.6 × 250 mm i.d. 5 μm) and a diode array detector (DAD, Agilent Technologies, Palo Alto, CA, USA). The mobile phase was a mixture of phosphate buffer solution (0.1 M, pH = 6.7) and acetonitrile (83 : 17, v/v). The flow rate and the wavelength of DAD were set at 1.0 mL min^−1^ and 245 nm, respectively.

#### 2.3.4. Fourier transform infrared spectroscopy analysis

The Fourier transform infrared (FT-IR) spectra of JHP-1, JHP-2, JHP-3, JHP-4, and JHP-5 were measured using a Nicolet iS 10 FT-IR (ThermoFisher scientific, Waltham, MA, USA) based on our previously reported method.^10^

Furthermore, the esterification degree (DE) of JHPs was also determined from FT-IR spectra according to previously reported methods.^29,30^ The determination of DE was based on the band areas at 1700–1750 cm^−1^ (esterified uronic acids) and 1600–1630 cm^−1^ (free uronic acids). Afterwards, the DE was calculated according to the equation as follows:DE (%) = A1740/(A1740 + A1610) × 100

### 2.4. Evaluation of *in vitro* antioxidant activities of JHPs

The ABTS, DPPH, nitric oxide (NO), and hydroxyl (OH) radical scavenging activities of JHP-1, JHP-2, JHP-3, JHP-4, and JHP-5 were measured according to our previously reported methods.^10,31^ The ABTS, DPPH, NO, and OH radical scavenging activities of JHPs were measured at five different concentrations ranged from 0.1 mg mL^−1^ to 5.0 mg mL^−1^, from 0.1 mg mL^−1^ to 5.0 mg mL^−1^, from 0.35 mg mL^−1^ to 5.0 mg mL^−1^, and from 0.5 mg mL^−1^ to 5.0 mg mL^−1^, respectively, and the IC_50_ values (mg mL^−1^) of JHPs were calculated based on a logarithmic regression curve. The distilled water was used as a blank control, and the BHT and vitamin C were used as positive controls.

### 2.5. Evaluation of *in vitro* antiglycation activities of JHPs from different chrysanthemum teas

The BSA-Glucose model (BSA-Glc) was performed for the evaluation of antiglycation activity according to a previously reported method with minor modifications.^32^ The total 10 mL of reaction mixture consisted of BSA (20 mg mL^−1^), glucose (500 mM L^−1^), sodium azide (1%), phosphate buffer (200 mM L^−1^, pH 7.4), and each sample with different concentrations (0.25, 0.5, 1.0 and 2.0 mg mL^−1^). Aminoguanidine (AG) was used as the positive control. Then, the mixture was incubated at 37 °C for 14 days. The 1.0 mL of glycated solution was taken out from the whole mixture, and determined at an excitation/emission wavelength of 370/440 nm, which is characteristic of AGEs. The antiglycation activity (%) was calculated as the following equation below. The antiglycation activity was measured at four different concentrations, and a logarithmic regression curve was established to calculate IC_50_ values (mg mL^−1^).Antiglycation activity (%) = (1 − *F*_sample_/*F*_blank_) × 100%where *F*_sample_ is the fluorescence intensity of the mixture of the sample, the BSA, the glucose, and the sodium azide; and *F*_blank_ is the fluorescence intensity of the mixture of deionized water, the BSA, the glucose, and the sodium azide.

### 2.6. Statistical analysis

All experiments were conducted in triplicate, and data were expressed in means ± standard deviations. Statistical analysis was performed using Origin 9.0 software (OriginLab Corporation, Northampton, Mass., USA). Statistical significances were carried out by one-way analysis of variance (ANOVA), taking a level of *p* < 0.05 as significant to Duncan's multiple range test.

## 3. Results and discussions

### 3.1. Chemical compositions of JHPs

The extraction yields and chemical compositions of JHPs from different chrysanthemum teas are summarized in Table 1. As shown in Table 1, the extraction yields of JHP-1, JHP-2, JHP-3, JHP-4, and JHP-5 ranged from (3.2 ± 0.2)% to (7.3 ± 0.2)%, which is similar with previous studies.^10,23,33^ Indeed, the highest extraction yield of JHP-2 was observed among five JHPs extracted from different chrysanthemum teas. Results suggested that the different species and cultivars of chrysanthemum teas significantly affected the extraction yield of JHPs. Moreover, the total contents of polysaccharide in JHP-1, JHP-2, JHP-3, JHP-4, and JHP-5 ranged from (78.8 ± 0.2)% to (89.2 ± 0.2)%, which indicated that polysaccharides were the major biological components in JHPs. In contrast, a few proteins were detected in JHP-1, JHP-2, JHP-3, JHP-4, and JHP-5, which ranged from (1.1 ± 0.2)% to (4.2 ± 0.3)%. Furthermore, the contents of total uronic acids in JHP-1, JHP-2, JHP-3, JHP-4, and JHP-5 were determined to be (31.8 ± 0.1)%, (30.2 ± 0.2)%, (33.4 ± 0.2)%, (28.4 ± 0.3)%, and (36.2 ± 0.2)%, respectively. The relatively high uronic acids contents in JHPs suggested that pectin-like acidic polysaccharides existed in chrysanthemum teas.^10,11,27^ Moreover, although phenolic compounds were thoroughly removed by ethanol extraction, ethanol precipitation, and dialysis, a few phenolic compounds were also observed in JHP-1, JHP-2, JHP-3, JHP-4, and JHP-5. The contents of total phenolics in JHPs ranged from 9.4 ± 0.7 mg GAE per g to 70.2 ± 1.7 mg GAE per g, which suggested that natural polyphenols–polysaccharide conjugates might exist in JHPs extracted from chrysanthemum teas.^34–36^ Generally, the polyphenols can bind spontaneously to the plant cell-wall pectins during food processing through non-covalent interactions, such as hydrophobic interactions, hydrogen bonding, and ionic interactions.^37,38^ Phenolic compounds from natural resources usually exhibit strong antioxidant activity. The conjugation of phenolic compounds may improve the antioxidant effects of polysaccharides.^39,40^ The relatively high content of total phenolics determined in JHP-1 might contribute to its high antioxidant and antiglycation activities.

**Table 1:** Extraction yields and chemical compositions of JHP-1, JHP-2, JHP-3, JHP-4, and JHP-5 from different chrysanthemum teas^a^

	Chrysanthemum tea polysaccharides				
	JHP-1	JHP-2	JHP-3	JHP-4	JHP-5
Extraction yields (%)	4.1 ± 0.2^c^	7.3 ± 0.2^a^	3.2 ± 0.2^b^	4.2 ± 0.2^c^	5.5 ± 0.2 ^b^
Total sugars (%)	78.8 ± 0.2^d^	84.3 ± 0.2^c^	83.2 ± 0.2^c^	89.2 ± 0.2^a^	87.2 ± 0.1 ^b^
Uronic acids (%)	31.8 ± 0.1^c^	30.2 ± 0.2^c^	33.4 ± 0.2^b^	28.4 ± 0.3^d^	36.2 ± 0.2 ^a^
Proteins (%)	4.2 ± 0.2^a^	4.2 ± 0.1^a^	2.3 ± 0.1^c^	1.1 ± 0.2^d^	2.7 ± 0.1 ^b^
DE (%)	7.3 ± 0.1^e^	25.0 ± 0.1^d^	34.1 ± 0.1^c^	41.9 ± 0.2^b^	50.1 ± 0.2 ^a^
TPC (mg GAE per g)	70.2 ± 1.7^a^	12.8 ± 0.5^c^	14.9 ± 0.4^b^	9.4 ± 0.7^d^	10.4 ± 0.2 ^d^

a JHP-1, *Coreopsis tinctoria* tea polysaccharides; JHP-2, *Chrysanthemum indicum* tea polysaccharides; JHP-3, *C. morifolium* ‘Huangju’ tea polysaccharides; JHP-4, *C. morifolium* ‘Gongju’ tea polysaccharides; JHP-5, *C. morifolium* ‘Hangbaiju’ tea polysaccharides; DE, degree of esterification; TPC, content of total phenolics; values represent mean ± standard deviation, and superscripts a–e differ significantly (*p* < 0.05) among JHP-1, JHP-2, JHP-3, JHP-4 and JHP-5; statistical significances were carried out by ANOVA, followed by Duncan's test.

### 3.2. Molecular weights and constituent monosaccharides of JHPs

**Fig. 1:**
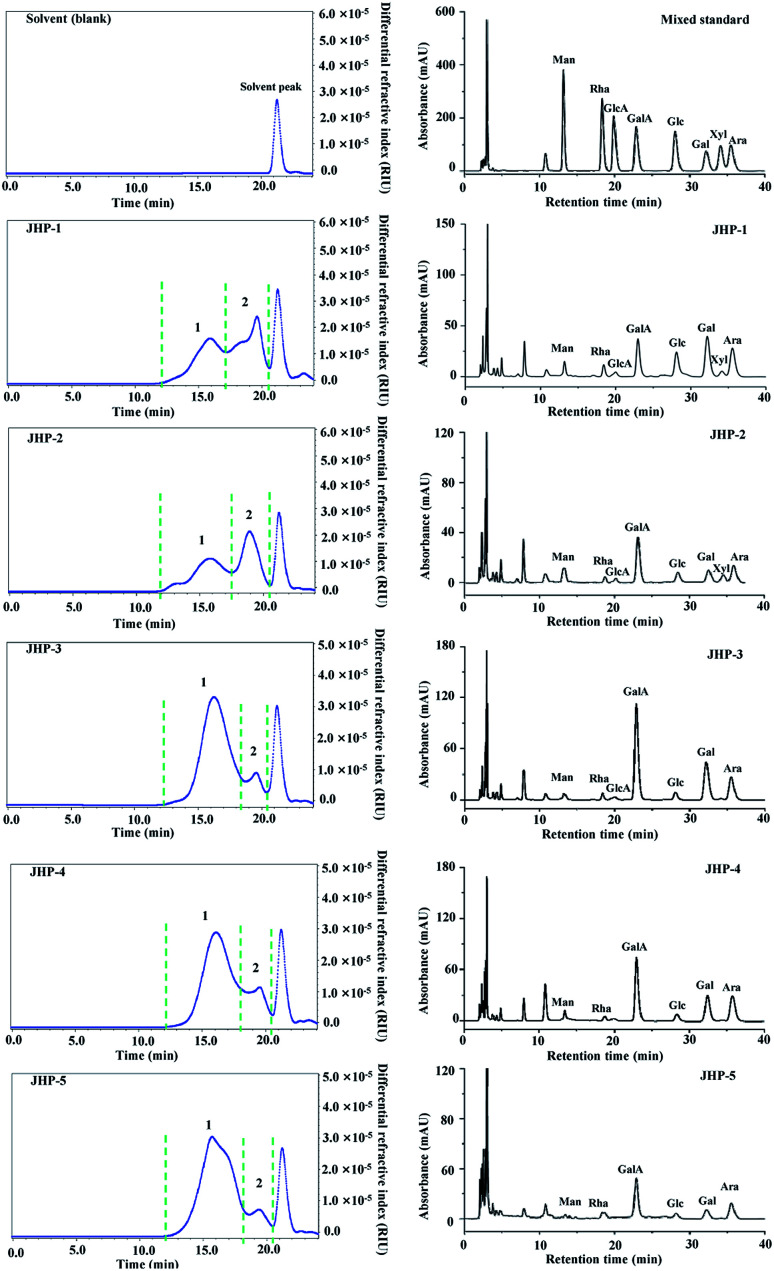
High performance size exclusion chromatograms (left) and high performance liquid chromatography profiles (right) of JHP-1, JHP-2, JHP-3, JHP-4, and JHP-5 from different chrysanthemum teas. JHP-1, *Coreopsis tinctoria* tea polysaccharides; JHP-2, *Chrysanthemum indicum* tea polysaccharides; JHP-3, *C. morifolium* ‘Huangju’ tea polysaccharides; JHP-4, *C. morifolium* ‘Gongju’ tea polysaccharides; JHP-5, *C. morifolium* ‘Hangbaiju’ tea polysaccharides; Gal, galactose; GalA, galacturonic acid; Ara, arabinose; Glc, glucose; Rha, rhamnose; Man, mannose; GlcA, glucuronic acid; Xyl, xylose.

Generally, it is considered that bioactivities of natural polysaccharides are closely correlated to their molecular weights and constituent monosaccharides.^41^ Therefore, molecular weights and constituent monosaccharides of JHPs extracted from different chrysanthemum teas were investigated and compared. Fig. 1 showed the HPSEC-RID chromatograms and HPLC-UV profiles of JHP-1, JHP-2, JHP-3, JHP-4, and JHP-5, respectively. Briefly, as shown in Fig. 1, besides the solvent peak (ranged from 20 min to 22 min), there were two polysaccharide fractions (fraction 1 and fraction 2) determined in JHP-1, JHP-2, JHP-3, JHP-4, and JHP-5, respectively. The HPSEC-RID chromatograms of JHP-3, JHP-4, and JHP-5 extracted from different cultivars of *C. morifolium* were similar (fraction 1 was the dominant peak, Fig. 1), but different from that of JHP-1 and JHP-2 (both fraction 1 and fraction 2 were the dominant peaks, Fig. 1). Results suggested that different species of chrysanthemum teas affected the molecular weight distributions of JHPs. The detailed molecular weights and molecular weight distributions of fraction 1 and fraction 2 in JHPs are summarized in Table 2. As shown in Table 2, the molecular weights of fraction 1 and fraction 2 in JHP-1, JHP-2, JHP-3, JHP-4, and JHP-5 ranged from 4.29 × 10^5^ Da to 5.88 × 10^5^ Da, and from 4.11 × 10^4^ Da to 5.24 × 10^4^ Da, respectively, which are similar with previous studies.^10,42,43^ The highest molecular weight of fraction 1 was measured in JHP-5 among all tested JHPs, and the lowest molecular weight was observed in JHP-1. The low molecular weights of natural polysaccharides may contribute to their relatively high antioxidant effects *in vitro*.^10,11,27^ Furthermore, the polydispersities of fraction 1 and fraction 2 in JHPs ranged from 1.63 to 1.92, and from 1.02 to 1.23, respectively. Results showed that the polysaccharide fraction 1 in JHPs possessed a relatively wide molecular weight distribution, while the polysaccharide fraction 2 in JHPs had a relatively narrow molecular weight distribution.

**Table 2:** Molecular weights (*M*_w_), polydispersity (*M*_w_/*M*_n_), and constituent monosaccharides of JHP-1, JHP-2, JHP-3, JHP-4, and JHP-5 from different chrysanthemum teas^a^

	Chrysanthemum tea polysaccharides				
	JHP-1	JHP-2	JHP-3	JHP-4	JHP-5
***M***_**w**_ **(Da)**					
Fraction 1, ×10^5^	4.29 (±0.03)^d^	4.44 (±0.03)^c^	5.42 (±0.12)^b^	5.30 (±0.05)^b^	5.88 (±0.08)^a^
Fraction 2, ×10^4^	5.24 (±0.09)^a^	4.91 (±0.12)^b^	4.26 (±0.07)^d^	4.11 (±0.11)^d^	4.55 (±0.06)^c^

***M***_**w**_/***M***_**n**_					
Fraction 1	1.89	1.92	1.80	1.88	1.63
Fraction 2	1.05	1.23	1.04	1.04	1.02

**Constituent monosaccharide and molar ratios**					
Galactose	1.00	1.00	1.00	1.00	1.00
Galacturonic acid	0.94	3.04	2.01	1.86	3.65
Arabinose	0.73	1.67	0.65	1.22	2.22
Glucose	0.68	0.62	0.18	0.20	0.39
Rhamnose	0.17	0.26	0.09	0.08	0.09
Mannose	0.15	0.61	0.02	0.21	0.08
Glucuronic acid	0.10	0.28	0.08	—	—
Xylose	0.10	0.43	—	—	—

a JHP-1, *Coreopsis tinctoria* tea polysaccharides; JHP-2, *Chrysanthemum indicum* tea polysaccharides; JHP-3, *C. morifolium* ‘Huangju’ tea polysaccharides; JHP-4, *C. morifolium* ‘Gongju’ tea polysaccharides; JHP-5, *C. morifolium* ‘Hangbaiju’ tea polysaccharides.

Furthermore, Fig. 1 also showed the HPLC-UV profiles of JHP-1, JHP-2, JHP-3, JHP-4, and JHP-5. Results showed that the HPLC-UV profiles of JHP-1 and JHP-2 were similar, but different from that of JHP-3, JHP-4, and JHP-5 extracted from *C. morifolium*. Results suggested that different species of chrysanthemum teas also affected the constituent monosaccharides of JHPs. In brief, the constituent monosaccharides of JHP-1 and JHP-2 were measured as Gal, GalA, Ara, Glc, Rha, Man, GlcA, and Xyl. The molar ratios of Gal, GalA, Ara, Glc, Rha, Man, GlcA, and Xyl in JHP-1 and JHP-2 were determined to be about 1.00 : 0.94 : 0.73 : 0.68 : 0.17 : 0.15 : 0.10 : 0.10, and 1.00 : 3.04 : 1.67 : 0.62 : 0.26 : 0.61 : 0.28 : 0.43, respectively (Table 2). The major constituent monosaccharides of JHP-3, JHP-4, and JHP-5 were measured as Gal, GalA, Ara, Glc, Rha, and Man. The molar ratios of Gal, GalA, Ara, Glc, Rha, and Man in JHP-3, JHP-4, and JHP-5 were determined to be about 1.00 : 2.01 : 0.65 : 0.18 : 0.09 : 0.02, 1.00 : 1.86 : 1.22 : 0.20 : 0.08 : 0.21, and 1.00 : 3.65 : 2.22 : 0.39 : 0.09 : 0.08, respectively. Results showed that GalA, Ara, and Gal were the dominant monosaccharides in JHPs extracted from different chrysanthemum teas, which further confirmed that JHPs were pectic-polysaccharides.^33,43^ Furthermore, rhamnogalacturonan I (RG I), homogalacturonan (HG), and arabinogalactan (AG II) might exist in JHPs from different chrysanthemum teas based on the constituent monosaccharides determined in JHPs.^10,33,42,43^

### 3.3. FT-IR spectra and degree of esterification of JHPs from different chrysanthemum teas

**Fig. 2:**
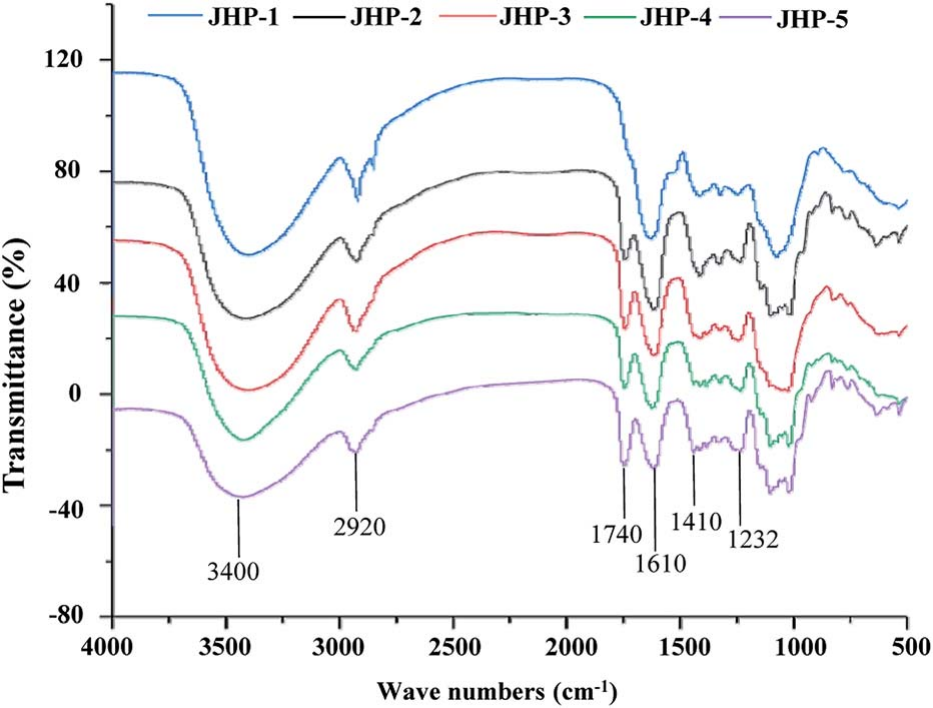
FT-IR spectra of JHP-1, JHP-2, JHP-3, JHP-4, and JHP-5 from different chrysanthemum teas. JHP-1, *Coreopsis tinctoria* tea polysaccharides; JHP-2, *Chrysanthemum indicum* tea polysaccharides; JHP-3, *C. morifolium* ‘Huangju’ tea polysaccharides; JHP-4, *C. morifolium* ‘Gongju’ tea polysaccharides; JHP-5, *C. morifolium* ‘Hangbaiju’ tea polysaccharides.

The FT-IR spectra were used for the determination of structural characteristics of JHPs from different chrysanthemum teas. Fig. 2 showed the FT-IR spectra of JHP-1, JHP-2, JHP-3, JHP-4, and JHP-5. As shown in Fig. 2, the FT-IR spectra of JHP-1, JHP-2, JHP-3, JHP-4, and JHP-5 were similar, which indicated that JHPs extracted from different chrysanthemum teas possessed similar chemical features. In brief, the intense and broad bands around 3200 cm^−1^ and 3600 cm^−1^ are the characteristic bands of hydroxyl groups.^10,27^ Bands in the region of 3000–2800 cm^−1^ are assigned to C–H absorption that includes –CH, –CH_2_, and –CH_3_ stretching vibrations.^27^ The absorption band at 1740 cm^−1^ is assigned to the C=O stretching vibration of esterified groups.^10^ Furthermore, the intense peak that appeared at 1610 cm^−1^ is assigned to the C=O asymmetric stretching of COO–, suggesting the existence of uronic acids.^27^ The band at 1410 cm^−1^ is due to the bending vibration of C–H or O–H.^44^ Typical bands of protein at 1651 cm^−1^ and 1555 cm^−1^ were not detected, which indicated the low amount of protein in JHPs (Table 1). Furthermore, the degree of esterification (DE) of JHPs extracted from different chrysanthemum teas was also investigated by FT-IR spectroscopy analysis. As shown in Table 1, the DE of JHPs extracted from different chrysanthemum teas ranged from 7.3% to 50.1%. The highest DE value (50.1%) was observed in JHP-5 among all tested JHPs, and the lowest DE value (7.3%) was observed in JHP-1. Previous studies have indicated that the DE value of pectic-polysaccharides was negative correlated to their antioxidant activities.^10,11,27^

### 3.4. *In vitro* antioxidant activities of JHPs

**Fig. 3:**
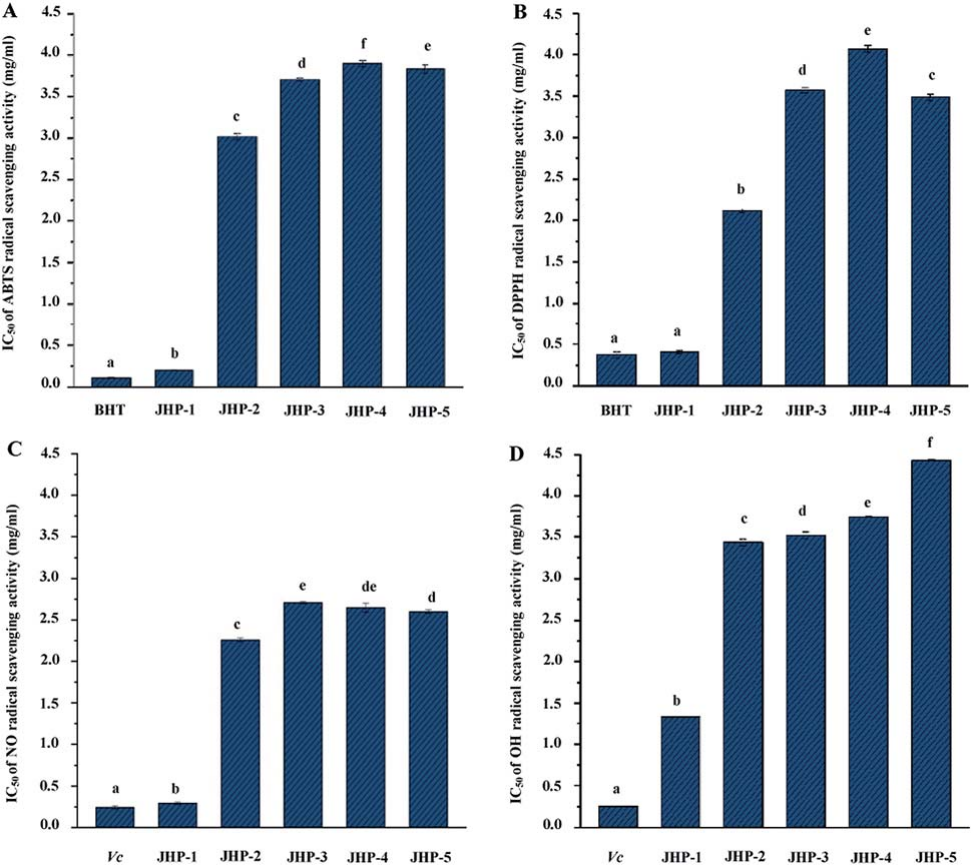
ABTS (A), DPPH (B), nitric oxide (C), and hydroxyl (D) radical scavenging activities of JHP-1, JHP-2, JHP-3, JHP-4, and JHP-5 from different chrysanthemum teas. JHP-1, *Coreopsis tinctoria* tea polysaccharides; JHP-2, *Chrysanthemum indicum* tea polysaccharides; JHP-3, *C. morifolium* ‘Huangju’ tea polysaccharides; JHP-4, *C. morifolium* ‘Gongju’ tea polysaccharides; JHP-5, *C. morifolium* ‘Hangbaiju’ tea polysaccharides; BHT, butylated hydroxytoluene; Vc, vitamin C; the error bars are standard deviations; significant (*p* < 0.05) differences are shown by data bearing different letters (a–f); statistical significances were carried out by ANOVA and Ducan's test.

Previous studies have shown that polysaccharides from chrysanthemum teas possess remarkable antioxidant activities.^10,23^ However, comparison of antioxidant activities of polysaccharides in different chrysanthemum teas has seldom been performed. The ABTS, DPPH, nitric oxide, and hydroxyl radical scavenging activities of JHP-1, JHP-2, JHP-3, JHP-4, and JHP-5 are shown in Fig. 3, respectively. Results showed that JHPs extracted from different chrysanthemum teas exhibited remarkable antioxidant activities. Briefly, as shown in Fig. 3A, JHPs extracted from different chrysanthemum teas exerted ABTS radical scavenging activities. The IC_50_ values of ABTS radical scavenging activities of JHPs ranged from 0.20 mg mL^−1^ to 3.89 mg mL^−1^, which were similar with previous studies.^10^ Significant differences were observed among JHP-1, JHP-2, JHP-3, JHP-4, and JHP-5, which suggested that different species and cultivars of chrysanthemum teas affect the antioxidant activity of JHPs. The significantly highest ABTS radical scavenging activity was observed in JHP-1 among all tested JHPs, while the lowest ABTS radical scavenging activity was determined in JHP-4. Obviously, the order of ABTS scavenging activities of JHPs was JHP-1 > JHP-2 > JHP-3 > JHP-5 > JHP-4. Compared with the positive control (BHT, IC_50_ = 0.11 mg mL^−1^), JHPs still exhibited remarkable ABTS radical scavenging activities. In addition, the ABTS radical scavenging activity of JHP-1 was much higher than that of polysaccharides isolated from commonly consumed tea materials in China, such as *Lycium barbarum*,^45^ dark tea (Qingzhuan brick tea),^46^ puerh tea.^47^

As shown in Fig. 3B, JHPs extracted from different chrysanthemum teas also exerted DPPH radical scavenging activities. The IC_50_ values of DPPH radical scavenging activities of JHPs ranged from 0.41 mg mL^−1^ to 4.58 mg mL^−1^. The significantly highest DPPH radical scavenging activity was also observed in JHP-1 among all tested JHPs, while the lowest DPPH radical scavenging activity was determined in JHP-4. The order of DPPH scavenging activities of JHPs was JHP-1 > JHP-2 > JHP-5 > JHP-3 > JHP-4. Furthermore, compared with the positive control (BHT, IC_50_ = 0.38 mg mL^−1^), JHP-2, JHP-3, JHP-4, and JHP-5 exhibited moderate DPPH radical scavenging activities, while JHP-1 exhibited strong DPPH radical scavenging activity. The DPPH radical scavenging activity of JHP-1 was also much higher than that of polysaccharides isolated from *L. barbarum*,^45^ dark tea,^48^ puerh tea,^47^ and oolong tea.^49^

Moreover, as shown in Fig. 3C, JHPs extracted from different chrysanthemum teas also exerted nitric oxide radical scavenging activities. The IC_50_ values of nitric oxide radical scavenging activities of JHPs ranged from 0.29 mg mL^−1^ to 2.71 mg mL^−1^. The significantly highest nitric oxide radical scavenging activity was also observed in JHP-1 among all tested JHPs, while the lowest nitric oxide radical scavenging activity was determined in JHP-3. Results further confirmed that JHP-1 exhibited remarkable antioxidant activities. The order of nitric oxide scavenging activities of JHPs was JHP-1 > JHP-2 > JHP-5 > JHP-4 > JHP-3. Indeed, the nitric oxide radical scavenging activity of JHP-1 was extremely close to that of vitamin C (IC_50_ = 0.24 mg mL^−1^), which indicated that JHP-1 exhibited strong nitric oxide radical scavenging activity. Furthermore, as shown in Fig. 3D, JHPs extracted from different chrysanthemum teas also exerted hydroxyl radical scavenging activities. The IC_50_ values of hydroxyl radical scavenging activities of JHPs ranged from 1.33 mg mL^−1^ to 4.42 mg mL^−1^. The order of hydroxyl scavenging activities of JHPs was JHP-1 > JHP-2 > JHP-3 > JHP-4 > JHP-5. Compared with the positive control (vitamin C, IC_50_ = 0.25 mg mL^−1^), all tested JHPs exhibited moderate hydroxyl radical scavenging activities. All results suggested that JHPs exhibited remarkable antioxidant activities *in vitro*, and JHPs could be one of the major contributors toward the antioxidant activities of chrysanthemum teas. Results suggested that JHPs, especially JHP-1 extracted from snow chrysanthemum tea (*C. tinctoria*), had potential applications in the pharmaceutical and health food industries.

Generally, the antioxidant activities of natural polysaccharides are closely correlated to their chemical characters, molecular weights, and compositional monosaccharides (uronic acids),^10,27,50^ as well as phenolic compounds that bonded on polysaccharides.^35,39,51^ In addition, it is estimated that presence of electrophilic groups like keto or aldehyde in acidic polysaccharides facilitates the liberation of hydrogen from O–H bond, and these groups can improve the radical scavenging activities.^52^ In the present study, the highest antioxidant activities (ABTS, DPPH, nitric oxide, and hydroxyl radical scavenging activities) observed in JHP-1 among all tested JHPs might be partially attributed to its relatively low molecular weight, high content of unmethylated uronic acids, and high content of conjugated phenolics as abovementioned. Previous studies have also shown that polysaccharides with low molecular weight and high content of unmethylated uronic acids exert high antioxidant activities.^10,27,53^ Additionally, previous study has shown that phenolic compounds in chrysanthemum teas exert significant antioxidant activity.^15^ Generally, the conjugation of polyphenolics can significantly improve the antioxidant activities of polysaccharides.^39,54,55^ JHP-1 with the highest phenolic content exhibited the highest antioxidant activity, suggesting the polyphenolics may contribute to the antioxidant activity of JHPs.^56^ However, the further purification, structural characterization, and evaluation of antioxidant activities of JHPs and their different fractions are required to reveal their structure–bioactivity relationships.

### 3.5. *In vitro* antiglycation activities of JHPs

**Fig. 4:**
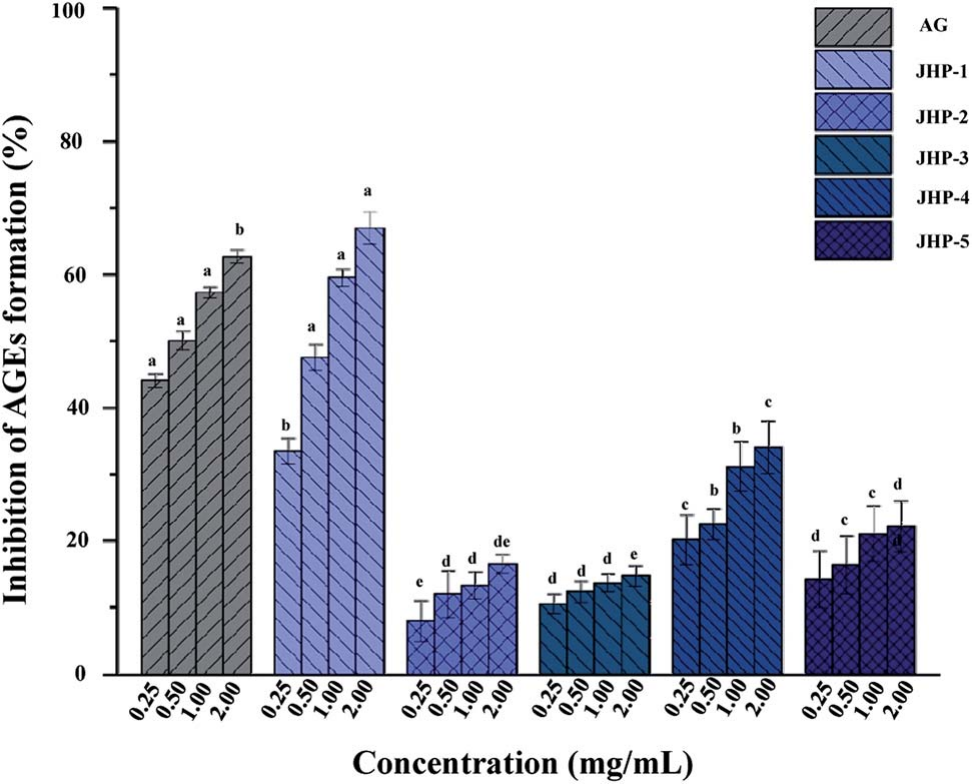
Antiglycation activities of JHP-1, JHP-2, JHP-3, JHP-4, and JHP-5 from different chrysanthemum teas. JHP-1, *Coreopsis tinctoria* tea polysaccharides; JHP-2, *Chrysanthemum indicum* tea polysaccharides; JHP-3, *C. morifolium* ‘Huangju’ tea polysaccharides; JHP-4, *C. morifolium* ‘Gongju’ tea polysaccharides; JHP-5, *C. morifolium* ‘Hangbaiju’ tea polysaccharides.

Recently, several studies have revealed that the formation of AGEs, are thought to contribute to the development of diabetes mellitus and its complications,^57^ and compounds with combined antioxidant and antiglycation properties are more efficient in diabetes mellitus treatment.^4,57,58^ This study has shown that JHPs exhibited remarkable antioxidant activity. Thus, the antiglycation activity of JHPs was further investigated in the present study. The antiglycation activities of JHP-1, JHP-2, JHP-3, JHP-4, and JHP-5 are shown in Fig. 4, respectively. Results showed that the antiglycation activities of JHPs exhibited a dose-dependent manner. Briefly, the antiglycation activity of JHP-1 was also significantly higher than that of JHP-2, JHP-3, JHP-4, and JHP-5 at the concentrations of 0.25 to 2.0 mg mL^−1^, which is similar with the antioxidant activity of JHPs. Furthermore, the IC_50_ value of antiglycation activity of JHP-1 (0.61 mg mL^−1^) was extremely close to that of AG (the positive control, IC_50_ = 0.48 mg mL^−1^), which suggested that JHP-1 exhibited extremely strong antiglycation activity. Even at the concentration of 2.0 mg mL^−1^, the antiglycation activity of JHP-1 (66.9%) was much higher than that of the positive control (62.6%). Furthermore, the antiglycation activity of JHP-1 was also much higher than that of pectic-polysaccharides extracted from other plants, such as *Polygonum multiflorum* Thunb^57^ and *Actinidia argute*.^59^ Previous studies have shown that the mechanism of antiglycation may be related to its antioxidant activity.^9^ Results suggested that the high antiglycation activity of JHP-1 was positively correlated to their remarkable antioxidant activities, which might be partially attributed to its high content of unmethylated uronic acids and high content of conjugated phenolics as abovementioned.^4–6,60^

## 4. Conclusions

In this study, the physicochemical characteristics, antioxidant and antiglycation activities of JHPs extracted from different chrysanthemum teas were investigated and compared. Results showed that the molecular weights, molecular weight distributions, constituent monosaccharides, and degrees of esterification of JHPs were different, which are helpful for the better understanding of the chemical structures of JHPs extracted from chrysanthemum teas. Indeed, JHPs exhibited remarkable antioxidant activity and antiglycation activity, which indicated that JHPs could be one of the major contributors toward the antioxidant activities of chrysanthemum teas. Results suggested that JHPs, especially JHP-1 extracted from snow chrysanthemum tea, had good potential applications in the functional and health food industries.

## Conflicts of interest

The authors declare that there are no conflicts of interest.

## Supplementary Material
